# Effects of cigarette smoke on degranulation and NO production by mast cells and epithelial cells

**DOI:** 10.1186/1465-9921-6-108

**Published:** 2005-09-19

**Authors:** Xiu M Wei, Henry S Kim, Rakesh K Kumar, Gavin J Heywood, John E Hunt, H Patrick McNeil, Paul S Thomas

**Affiliations:** 1 Inflammation Research Unit, School of Pathology, Faculty of Medicine, UNSW, Sydney, Australia; 2 Department of Respiratory Medicine, Prince of Wales Hospital, Randwick, NSW, 2031, Australia

**Keywords:** nitric oxide, mast cells, epithelial cells, cigarette smoke

## Abstract

Exhaled nitric oxide (eNO) is decreased by cigarette smoking. The hypothesis that oxides of nitrogen (NO_X_) in cigarette smoke solution (CSS) may exert a negative feedback mechanism upon NO release from epithelial (AEC, A549, and NHTBE) and basophilic cells (RBL-2H3) was tested in vitro. CSS inhibited both NO production and degranulation (measured as release of beta-hexosaminidase) in a dose-dependent manner from RBL-2H3 cells. Inhibition of NO production by CSS in AEC, A549, and NHTBE cells was also dose-dependent. In addition, CSS decreased expression of NOS mRNA and protein expression. The addition of NO inhibitors and scavengers did not, however, reverse the effects of CSS, nor did a NO donor (SNP) or nicotine mimic CSS. N-acetyl-cysteine, partially reversed the inhibition of beta-hexosaminidase release suggesting CSS may act via oxidative free radicals. Thus, some of the inhibitory effects of CSS appear to be via oxidative free radicals rather than a NO_X _-related negative feedback.

## Introduction

Cigarette smoke is a complex medium containing approximately 4000 different constituents [1] separated into gaseous and particulate phases. The components of the gaseous phase include carbon monoxide, carbon dioxide, ammonia, hydrogen dioxide, hydrogen cyanide, volatile sulphur-containing compounds, nitrogen oxides (including nitric oxide, NO), and other nitrogen-containing compounds. The particulate phase contains nicotine, water and tar [2]. Pulmonary effects of cigarette smoke include chronic obstructive pulmonary disease, increased airway reactivity, exacerbations of asthma, and an increased frequency of pulmonary infections. These effects are considered to be due to the direct actions of cigarette-derived toxins and ciliotoxins causing connective tissue destruction, hypersecretion, pooling of mucus and blebbing of membranes of endothelial cells. Cigarette smoke also reduces levels of exhaled nitric oxide in active and passive smokers, suggesting that it inhibits NO production [3-5]. Su et al [6] have shown that exposure to cigarette smoke extract inhibits the activity, protein and messenger RNA of NO synthase (eNOS) in pulmonary artery endothelial cells irreversibly. Whether alterations in NO play a role in the increased risk of pulmonary disease is not completely understood.

Mast cells play a crucial role in acute and allergic inflammation, and have high-affinity receptors for IgE (FcεRI) on their surface. Cross-linking of surface IgE molecules results in exocytosis of preformed mediators such as amines and proteases, as well as release of newly generated mediators including leukotrienes, prostaglandins and a variety of cytokines [7]. In the lungs and skin of smokers mast cells increase in absolute numbers and smoking may be associated with activation of mast cells [8,9]. They may contribute to some of the changes seen in smoking by releasing chemotactic factors, secreting proteases and other mediators. Some reports suggest that NO may be a participant in mast cell activation, but others suggest that it may also inhibit mast cell pre-formed mediator release [10,11]. Since cigarette smoke contains high levels of NO, it was hypothesised that NO may exert an inhibitory effect on degranulation, perhaps via negative feedback.

Airway epithelial cells (AEC) are important regulators of inflammation in the airway [12]. They have a function in host defence and play a significant role in airway inflammation by releasing NO, a potentially important mediator of airway inflammation [13,14], as well as releasing other mediators and recruiting inflammatory cells [12,15,16]. Cigarette smoke interferes with and inhibits the normal function of AEC by a variety of mechanisms. Some of these include decreases in the level of exhaled NO, enhanced release of pro-inflammatory cytokines, and inhibition of the airway repair process [5,17,18].

This study was designed to examine whether cigarette smoke induces dysfunction of airway mast cells and epithelial cells via the donation of cigarette-derived NO. It was hypothesized that the NO from cigarette smoke may induce negative feedback and cause a reduction in endogenous NO production from mast cells and epithelial cells. Thus, NO scavengers were added to a cigarette smoke solution (CSS). In addition, a NO donor was studied as a positive control and NO inhibitors as controls for endogenous NO production. NO generation was measured as nitrite.

A rat basophilic leukemia cell line, RBL-2H3 representing mucosal type mast cells [19], which has been extensively applied in studies of mast cell biochemistry and signalling, was used as an in vitro model of mast cells for this study. Beta-hexosaminidase was used as a marker of mast cell activation and degranulation. Primary cultures of murine epithelial cells, normal human tracheobronchial (NHTBE) and transformed alveolar epithelial (A549) cell lines were studied in parallel [20,21].

## Materials and methods

Cell culture and polymerase chain reaction (PCR) reagents were purchased from Invitrogen Corporation (Sydney, Australia) and chemical reagents were bought from Sigma-Aldrich, (Sydney, Australia) unless otherwise specified. Animal tissue research was approved by the institutional animal ethics committee.

### Cell Culture

The rat basophilic leukemia cell line, RBL-2H3 (ATCC, American Type Culture Collection, Rockville, MD, USA) was grown in complete Eagle Minimal Essential Medium with 15% fetal bovine serum (FBS), 0.1 mM non-essential amino acids, 1.0 mM sodium pyruvate, 2.0 mM L-glutamine, 50 IU/ml penicillin and 50 μg/ml streptomycin. A549 cell line (ATCC) was maintained in complete F-12 Nutrient Medium supplemented with 10% FBS, 50 IU/ml penicillin and 50 μg/ml streptomycin. Mouse airway tracheal epithelial cells (AEC), obtained from tracheas of 8–10 week-old specific pathogen-free BALB/C, were cultured and maintained as previously described [20] on collagen-coated plastic ware. Third- to fifth-passage AEC were used for experiments. Normal human tracheal bronchial epithelial cells (NHTBE, Clonetics, USA) were maintained in Bronchial Epithelial Cell Growth Medium (BEGM) Bullet Kit (CC-3170, Clonetics, San Diego, CA, USA).

### Preparation of the cigarette smoke solution (CSS)

Water-soluble extract of cigarette smoke (both gas and particulate phases) was prepared as described previously [22]. Briefly, mainstream smoke from commercial cigarettes (Marlboro, Philip Morris, Australia) was drawn through 1 ml of medium by the application of a vacuum to the vessel containing the medium. Each cigarette was burned for 5 min, and 5 cigarettes were used for each millilitre of the appropriate medium for different cells. The pH of the resultant extract was titrated to pH 7.4, and diluted with medium. Solutions ranging from 0.125% to 1.0% were used in the present study in response to preliminary experiments which indicated that these were non-toxic concentrations. CSS was used within 2 hrs of preparation, and the NOx content of the CSS was in the range 1.3–2.6 mM, mean 1.76 (S.E. 0.67) mM. CSS was incubated also in control wells with media but without cells at the same concentrations and for the time periods. The final NOx content in these latter wells was subtracted from the values in the experimental wells.

### Beta-hexosaminidase secretion assay

10^6^/ml RBL-2H3 cells were sensitised with 100 ng/ml of mouse monoclonal IgE anti-DNP overnight. Cells were washed twice with phosphate buffered saline (PBS) and pre-incubated with different concentrations of CSS for a further 6 h prior to activation with either 100 ng/ml DNP-HSA antigen or 10 μmol/L of calcium ionophore A23187. Beta-hexosaminidase release from RBL-2H3 was measured by incubating 25 μl of the supernatant or lysed cell pellet with 5% Triton-100 with 25 μl of p-NAG in a 96-well plate (Nunc, Roskilde, DM) for 2 h at 37°C. The reaction was stopped with 250 μl 0.2 M glycine (pH 10.6) and the resultant change in absorbance read at 405 nm. The net percentage of release of beta-hexosaminidase was calculated by the following formula:

net percent release (%) = [S/(S+P)-S_control_/(S_control_+P_control_)] × 100,

where S, P are the mediator contents of supernatants and pellets of stimulated cells, respectively, S_control_/(S_control_+P_control_)(%) is spontaneous release of mediator without a stimulus.

### Nitrite and nitrate measurements

RBL-2H3, A549, NHTBE and AEC were cultured in complete media until 90% confluent. Cells were washed with PBS and incubated with nicotine (31.25 ng/ml–400 ng/ml) or CSS as above for a further 24 h or 48 h (A549 cells), then measured as nitrite and nitrate (NOx) accumulation in media as described previously [23-25]. Briefly, nitrate was measured as nitrite after enzymatic conversion by nitrate reductase. Volumes of 20 μl NADPH, 10 μl FAD and 20 μl nitrate reductase were diluted in reaction buffer and added to yield final concentrations of 50 μmol/L, 5 μmol/L and 200 IU/L, respectively. Samples of 50 μl each were subsequently incubated for 1 hour at 37°C. Next, 10 μl of 2,3-diaminonaphthalene (DAN, 0.05 mg/ml in 0.62 M HCl) was added to each well and incubated for an additional 10 mins. The reaction was stopped by 10 μl of 2.8 M NaOH. The fluorescence of final product (1*H*-naphthotriazole) was measured using Perkin-Elmer Cytofluor 4000 plate reader (excitation 360/40, emission 395/25, gain 50). Nitrite concentration was calculated using a standard curve of serially diluted sodium nitrite.

### RT-PCR analysis of iNOS and eNOS expression

Cells were incubated with 1%CSS at different time-points (3 h, 6 h, 24 h). Total cellular RNA was extracted using TRI-Reagent (Sigma) according to the manufacturer's instructions. First-strand cDNA was synthesized from 1 μg total RNA with SuperScript II using Oligo (dT) as primers (Invitrogen, Carlsbad, CA, USA). PCR was performed on the reverse transcription products using specific oligonucleotide primers, and glyceraldehyde-3-phosphate dehydrogenase (GAPDH) was used as a housekeeping gene.

PCR reactions contained 2 μL cDNA, 10 μM primers (Table 1), 1.5 mmol/L Mg, 200 μmol/L dNTPs and 0.5 IU Platinum Taq polymerase (Invitrogen) in a total reaction volume of 50 μL. PCR products were electrophoresed on 1.2% agarose gel containing 0.1% ethidium bromide. Positive and negative controls were run concurrently to exclude DNA contamination.

**Table 1:** PCR primers as used in Methods.

NOS isoform	Primer sequence
Rat iNOS [26]	sense:5'-GGACCACCTCTATCAGGAA-3', antisense 5'-CCTCATGATAACGTTTCTGGC-3';
Rat eNOS [27]	sense: 5'-TACCAGCCGGGGGACCAC-3', antisense: 5'-CGAGCTGAC-AGAGTAGTA-3'.
Human iNOS [28]	sense: 5'-GAGCTTCTACCT-CAAGCTATC-3', antisense: 5'-CCTGATGTTGCCATTGTTGGT-3';
Human eNOS	sense:5'-GCACAGGAA-ATGTTCACC TAC-3', antisense: 5'-CACGATGGTGAC-TTTGGCTAG-3'.
Mouse iNOS (real time PCR) probe	sense: 5'-CAGCTGGGCTGTACAAACCTT-3', antisense: 5'-CATTGGAAGTGAAGCGTTTCG-3', 5'-6FAM (fluorescent reporter dye, 6-carboxyfluorescein)-CGGGCAGCCTGTGAGACCTTTGA-TAMRA (quenching agent, 6-carboxytetramethylrhodamine, Applied Biosystems, CA, USA).

#### Rat iNOS and eNOS conditions

After initial denaturation at 95°C for 2 min, 25–35 cycles of amplifications at 94°C 30 sec, 60°C for 30 sec and 72°C for 45 sec were carried out using Perkin-Elmer 2400 thermal cycler.

#### Human iNOS and eNOS conditions

The thermocycle consisted of 94°C for 30 sec, 58°C (eNOS)/60°C (i NOS and GAPDH) for 30 sec and 72°C for 45 sec. The numbers of amplification cycles were 28–35 (iNOS and eNOS) and 25 (GAPDH).

### Quantitative Real Time-PCR analysis

Due to the difficulty of growing large numbers of mouse AEC cells, quantitative real-time RT-PCR, which only requires small amounts of RNA, was chosen to determine mouse iNOS mRNA levels and β-actin (internal standard). Quantification of mRNA was performed by determining the threshold cycle (C_T_) on ABI PRISM 7700 Sequence Detector (Perkin-Elmer, Applied Biosystem). Standard curves were constructed using the values obtained from serially diluted positive control mouse iNOS plasmid.

Real time-PCR was performed in 50 μl reaction volumes containing 2X TaqMan Universal PCR Master Mix 25 μl (Roche, Branchburg, New Jersey, USA), 2.5 μl 18 μM sense/antisense primers, 2.5 μl 5 μM probe and 7 μl cDNA samples (Table 1). The following thermal profile was used: 2 min at 50°C, 10 min at 95°C and 50 cycles of 95°C for 15 sec, 60°C for 1 min.

### iNOS/eNOS Western-Blot

CSS-treated RBL-2H3 and A549 cells were rinsed with PBS and isolated by scraping in ice cold radio-immunoprecipitation (RIPA) buffer (1% NP-40, 0.5% sodium deoxycholate, 0.1% SDS in PBS) with freshly added aprotinin (30 μl/mL RIPA). Cell lysate was passed several times through a 25 gauge needle to shear the DNA and incubated 30 minutes on ice. RIPA (10 μl/ml) with 10 mg/ml phenylmethylsulfonylfluoride (PMSF) was added and cell lysate was microcentrifuged at 12,000 rpm for 20 minutes at 4°C. Protein concentration was determined using the Bradford method (Bio-Rad, Hercules, CA, USA). Supernatants (20 μg) were loaded on NuPAGE™ 4–12%Bis-Tris Gels (Invitrogen Corp.) and transferred to nitrocellulose membranes. The membranes were blocked overnight in 5% skimmed milk and incubated for 1 h at room temperature with primary antibodies at dilutions of 1:3000(iNOS/eNOS, BD Transduction Laboratories, San Diego, CA, USA). Peroxidase-labeled secondary antibody rabbit anti-mouse IgG was added at a dilution of 1:1000 (DAKO, CA, USA) for 1 h at RT. Membranes were developed with Enhanced Chemiluminescence Reagent (Perkin-Elmer Life Sciences, Boston, MA, USA) for 1 min and exposed for 30 sec to scientific imaging film (BioMax, Kodak, Rochester, USA).

### Flow cytometric analyses of iNOS/eNOS proteins

CSS (1%) was added to RBL-2H3 and A549 cells for 24 h, which were then trypsinized and fixed in 4% formaldehyde. Cells were permeabilized in 0.15% saponin PBS/2% BSA for 1 h on ice and washed in PBS/2% BSA. Subsequently, cells were incubated in 10 μg/mL anti-iNOS or 20 μg/mL anti-eNOS (BD Transduction Laboratories) for 30 mins on ice, washed twice and added goat anti-mouse IgG conjugated with FITC (DAKO) for further 30 mins in the dark on ice. Cells were washed, resuspended in 500 μl 1% formaldehyde, and analyzed by flow cytometry (BD FACSort). Mouse IgG1 and IgG2a (DAKO) were used as negative controls for iNOS and eNOS antibodies, respectively.

### Cell viability and cytotoxicity

Viability and cytotoxicity were assessed by trypan blue vital dye exclusion and lactate dehydrogenase release (LDH Kit, Sigma).

### Statistical analysis

Data are expressed as mean ± SEM. Analysis was performed by one-way ANOVA with the application of Dunnett's multiple comparisons test, and a p value <0.05 was considered significant. Data are representative of at least 3 different experiments. In the case of data expressed as percentage of baseline, the ANOVA and subsequent comparisons were performed on the raw data, prior to transformation.

## Results

### Effect of CSS on degranulation and [NO_X_] generation by RBL-2H3

**Figure 1:**
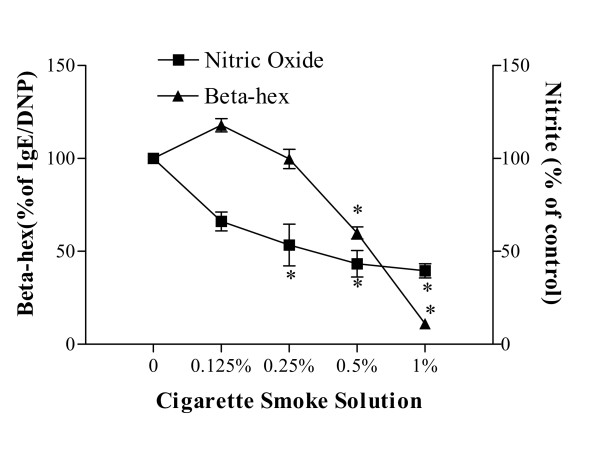
Effects of CSS on the release of beta-hexosaminidase and NOx production from RBL-2H3. Cells were passively sensitised with anti-DNP IgE, and incubated with 0.125–1% CSS for 6 hrs prior to activation with DNP. There is a CSS concentration-related decrease in the release of beta hexosaminidase. NOx formation was similarly decreased after 24 h incubation with the same range of CSS. Data are the means of 6 experiments with the mean and S.E.M. expressed as % of control, where baseline NOx = 3.04 +/- 0.21 μM, (ANOVA performed on raw data, *p < 0.001 with Dunnett's multiple comparison test compared to baseline values).

RBL-2H3 cells activated with either IgE/DNP or A23187 showed a concentration-dependent decrease in degranulation after incubation with CSS (0.125%–1.0%) for 6 h (Fig 1, p < 0.0001, ANOVA). Treatment with CSS decreased the percentage of beta-hexosaminidase release by up to 89% at 1.0% CSS. The CSS-induced inhibition of beta-hexosaminidase was also observed after 2 h incubation (data not shown). As shown in Figure 1, RBL-2H3 treated with CSS (0.125% to 1.0%) for 24 h resulted in a concentration-related inhibition of nitrite production (p < 0.05, ANOVA, where baseline NOx = 3.04 +/- 0.21 μM), with only a slight inhibition of nitrite at 6 h (p > 0.05, data not shown). Viability of RBL-2H3 and epithelial cells remained constant (generally >90%) before and after CSS treatment.

### Effect of nicotine on degranulation and [NO_X_] in RBL-2H3, A549, NHTBE, and airway epithelial cells (AEC)

**Figure 2:**
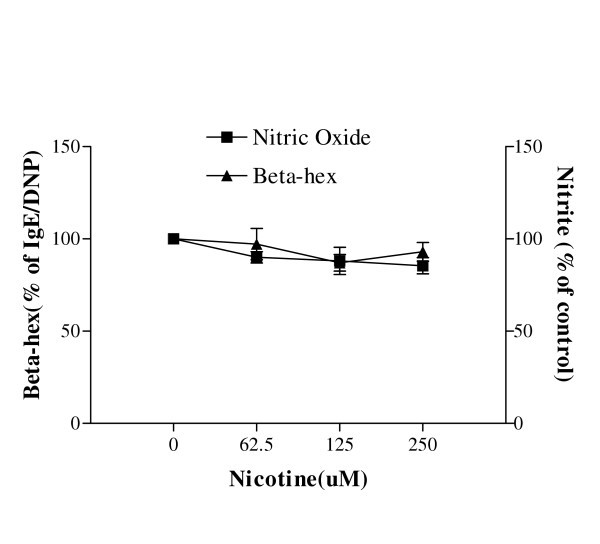
Effects of nicotine on the release of beta-hexosaminidase and NOx production from RBL-2H3. Cells were passively sensitised with anti-DNP IgE, incubated in nicotine solutions (62.5–250 μM) for 24 h, and then activated with DNP for beta hexosaminidae release, or accumulated NOx measured. No significant effects were seen. Data are the means of 4 experiments with the mean and S.E.M. expressed as % of control where mean (SD) baseline NOx = 3.5 +/- 0.11 μM (ANOVA performed on raw data, p > 0.05).

In order to investigate whether nicotine, the major component of cigarette smoking in particulate phase, is responsible for the inhibition of degranulation and production of nitric oxide, RBL-2H3 cells were incubated in nicotine solutions (62.5–250 μM) adjusted to physiological pH for 24 h. Neither degranulation nor production of nitric oxide was affected (Figure 2). Incubation of A549 cells with nicotine (31.25–400 ng/ml) for 48 h caused a significant decrease in production of NO_x_, but incubation of NHTBE and AEC cells with nicotine solutions (31.25 ng/ml–400 ng/ml) did not demonstrate any significant change (Table 2).

**Table 2:** Effects of nicotine on the production of NO, measured as NOx from A549, AEC, and NHTBE cell lines over 48 hrs. Data are expressed as mean (S.E.M.) % of control of 3 experiments (ANOVA performed on raw data, * p < 0.05 in A549 group; p > 0.05 in AEC and NHTBE groups; Dunnett's multiple comparison test)

NOx (Percentage of Control)				
Nicotine(μM)	31.25	62.5	125	250

A549 Cell Line	67.0 ± 5.79*	65.0 ± 7.87*	69.8 ± 7.12*	69.4 ± 7.81*
AEC Cells	112.8 ± 25.06	120.2 ± 9.70	105.7 ± 20.68	108.5 ± 22.95
NHTBE Cell Line	87.9 ± 8.61	83.4 ± 10.53	87.9 ± 3.37	99.9 ± 0.07

### Effects of NO pathway inhibitors on degranulation of RBL-2H3

To investigate whether the NO present in CSS may have a role in mast cell inhibition, a NO scavenger (hemoglobin) was pre-incubated for 1 h with CSS. A NO donor (SNP) was used as a positive control in studies without CSS. In addition, in case CSS stimulated the production of NO in these cells, an inhibitor of NOS (L-NMMA), and a cGMP inhibitor (ODQ) were studied in conjunction with CSS. The doses chosen were those optimised in previous work [29].

Activated or resting RBL-2H3 monolayers were incubated with: sodium nitroprusside (SNP 1 μM, 10 μM, and 100 μM); CSS plus hemoglobin (100 μM and 1 mM); or L-NMMA (1 μM, 10 μM and 100 μM) for 5 h. None of these was found to have a significant effect on basal or IgE mediated beta-hexosaminidase release, nor did hemoglobin or L-NMMA affect CSS-induced inhibition of beta-hexosaminidase release. Hemoglobin (1 mM) was pre-incubated with CSS for 1 h to neutralise NO in CSS and no effect was observed upon the degranulation of RBL-2H3. Pre-incubation of RBL-2H3 cells with the guanylyl cyclase inhibitor ODQ (10^-6^M) for 30 min did not reverse the CSS-induced inhibition of degranulation.

### Effects of CSS on [NO_X_] from AEC, A549 and NHTBE cells

**Figure 3:**
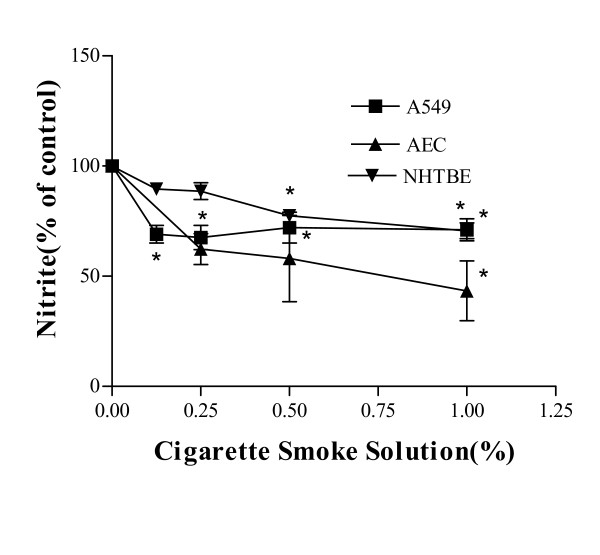
Effects of CSS on the production of NOx from A549, mouse AEC, and NHTBE. Cells were incubated with CSS for 24 h (AEC and NHTBE) or 48 h (A549). NOx production was assessed from the supernatants from each condition and cell line. Data are the means of 3 experiments with the mean and S.E.M. expressed as % of control. Baseline mean (SD) NOx of the cell lines were: A549 2.7 +/- 0.16 μM; mouse AEC 1.34 +/- 0.2 μM, and NHTBE 1.56 +/- 0.18 μM. ANOVA was performed on raw data: A549, *p < 0.05; AEC, *p < 0.05, 1% CSS compared with control; NHTBE, *p < 0.01, 1.0-0.5% CSS compared with control, *p < 0.05, 0.25% CSS vs. control; Dunnett's multiple comparison test.

Incubation of A549 cells with CSS (0.125%–1.0%) for 48 h caused a significant and dose-related decrease in production of NO_x _. Also, 24 h incubation of NHTBE and AEC with CSS (0.25%–1.0%) resulted in a dose-related inhibition of NO production, which ranged between 47 and 67% inhbition with 1% CSS (baseline mean (SD) NOx of the cell lines were: A549 2.7 +/- 0.16 μM; mouse AEC 1.34 +/- 0.2 μM, and NHTBE 1.56 +/- 0.18 μM, Figure 3).

### Expression of NOS isoforms

**Figure 4:**
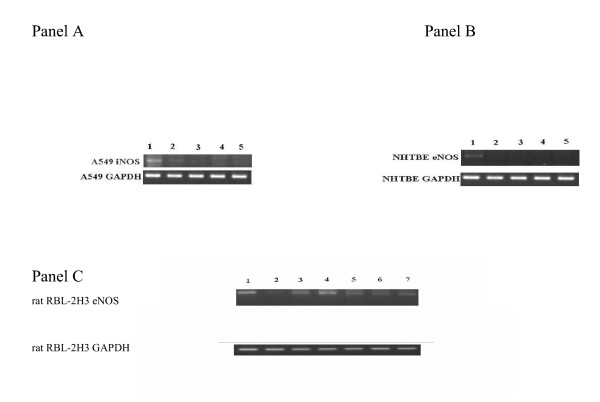
*RT-PCR analysis of NOS expression. ****Panel A***. Time course of the effect of 1% CSS upon A549 iNOS mRNA expression. Upper panel: Lane 1: control, Lane 2: 3 h, Lane 3: 6 h, Lane 4: 24 h, Lane 5: 24 h CSS exposure and then cells returned to normal media for further 24 h; bottom panel: GAPDH corresponding to each sample. PCR gels shown are representative of three separate experiments. There was a decrease in iNOS mRNA in A549 cells reaching undetectable levels at 6 h, which persisted to 24 hr, even after further incubation in normal culture media. ***Panel B***. Time course of the effect of 1% CSS on NHTBE eNOS mRNA expression. Upper panel: Lane 1: control, Lane 2: 3 h, Lane 3: 6 h, Lane 4: 24 h, Lane 5: 24 h CSS and returned to normal media for further 24 h; bottom panel: GAPDH corresponding to each sample. PCR gels are representative of three separate experiments. Similar changes were seen in the mRNA expression of eNOS in the NHTBE line as in the A549 cells with decreased levels at 3 h which persisted throughout the study period. ***Panel C***. Effect of 1% CSS and 100 μM nicotine on rat RBL-2H3 eNOS mRNA expression. Upper panel: Lane 1: control, Lane 2–4: CSS 3 h, 6 h, 24 h, Lanes 5–7: nicotine 3 h, 6 h, 24 h; bottom panel: GAPDH corresponding to each sample. PCR gels are representative of three separate experiments. After CSS, there was a decline in mRNA at 3 h which returned to baseline by 24 h, while exposure to nicotine showed a decrease at 6 and 24 h.

Incubation of A549 cells with 1% CSS at different time-points caused a time-dependent decrease in iNOS mRNA levels (Figure 4). No iNOS mRNA was detected after 6 h CSS incubation in this cell line. The same pattern was observed in NHTBE which only expressed eNOS mRNA. eNOS mRNA was not detected in A549 cells. In RBL-2H3 cells, the eNOS mRNA band decreased at 3 h, although it returned to control levels after 24 h. Nicotine treated RBL-2H3 cells resulted in a slight decrease in eNOS mRNA expression. There was no iNOS mRNA observed in this basophilic cell line.

### Quantitative Real Time-PCR analysis

**Figure 5:**
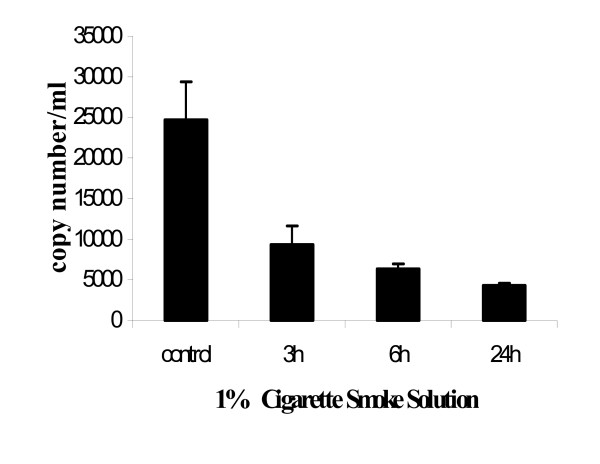
Effects of CSS on iNOS mRNA from mouse AEC by real-time PCR. Quantitative real-time RT-PCR was performed to determine mouse iNOS mRNA levels. Quantification of mRNA was performed by determining the threshold cycle and standard curves were constructed using the values obtained from serially diluted positive control mouse iNOS plasmid, and determining the values for experimental samples from these curves. A progressive fall in copy number was seen in AEC iNOS expression with 1% CSS, over 24 h. Data points represent 3 replicates with S.E.M.

Using the technique of real-time PCR to detect changes in AEC iNOS expression with 1% CSS, a progressive fall in copy number was seen over 24 h (Figure 5). The time-course is similar to that seen in the RT-PCR data for the A549 cells above.

### NOS protein expression by Western-Blot and flow cytometry

Using flow cytometry to detect iNOS positive cells, the number of cells expressing iNOS protein were seen to be decreased by 1% CSS in A549 cells. The ratio of positive to negative cells declined from 1.60 to 1.29 (t = 8.931, p = 0.012, paired t test, two-tailed). Similarly, eNOS positive RBL-2H3 cells were decreased after 1% CSS treatment from 2.78 to 2.24 ± 0.17. iNOS and eNOS protein levels were undetectable by immunoblot.

### Effects of NAC on CSS-induced inhibition of degranulation of RBL-2H3

**Figure 6:**
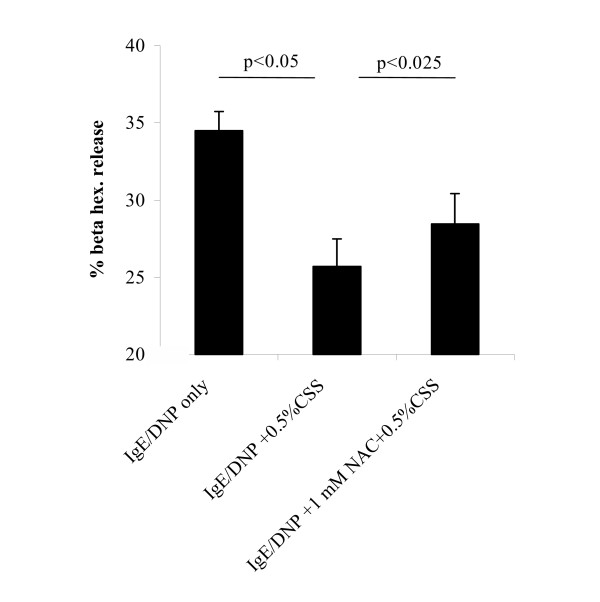
Effects of 1 mM NAC on 0.5 % CSS-induced inhibition of degranulation of RBL-2H3 cells. Data are the means of 5 experiments with the mean and S.E.M. expressed as % of total (ANOVA performed on raw data, Dunnett's multiple comparison test).

To investigate whether free radicals in CSS contribute to the inhibition of degranulation, RBL-2H3 cells were incubated with free radical scavenger N-acetyl-L-cysteine (NAC, 1 mM) for 30 min prior to their incubation with CSS. Compared with control CSS exposure and activation there was a significant reversal of the CSS-induced inhibition (Figure 6).

## Discussion

Nitric oxide is a ubiquitous intracellular and intercellular signaling molecule, which plays a role in the functions of various inflammatory cells including mast cells, lymphocytes, neutrophils and macrophages [30]. NO can have deleterious or beneficial roles in inflammatory conditions depending on the setting because of its role as both an immune mediator and an effector molecule [30]. There is increasing evidence that the interaction between NO and mast cells is important in the control of the human nasal airway response, the physiological and pathological regulation in immune system, and the inhibition of gastric acid secretion [30]. Some researchers have reported that NO may modulate mast cell pre-formed mediator release[31]. For instance, an increase in cGMP levels was found to inhibit histamine release in rat peritoneal mast cells which was reversed by L-NMMA (32). Brooks et al. [32] demonstrated that NO induced by interferon-gamma could inhibit the IgE-activated secretory function of mouse mixed peritoneal mast cells. Koranteng et al. [34] reported that NO generation inhibits pre-formed mediator release in murine peritoneal mast cells, but not in other mast cells which were of a different phenotype. CSS has variable effects upon isolated mast cells [22], but in vivo has been clearly demonstrated to reduce exhaled NO and the mechanism for this reduction was therefore studied in *in vitro *models of airway epithelial cells and mast cells.

The effects of cigarette smoke upon the NO pathways and NOS isoenzymes are controversial and may vary according to the disease, model or location of the NOS. For example, while exhaled NO has been shown to be decreased in humans after acute cigarette exposure, iNOS mRNA expression increased in the lungs of rats exposed to cigarette smoke, while nNOS showed a longer term increase in both transcription and translation [3,5,35,36]. Cigarette smoke has been shown, however, to cause a reduction in nitrite concentration and iNOS expression in a murine lung epithelial cell line in vitro[37]. In contrast, Comhair et al showed no change in iNOS expression in airway cells from healthy subjects exposed to cigarette smoke [38]. The effects of cigarette smoke on NOS in the vasculature has shown a reduction in ecNOS in the pulmonary vessels in vitro and in vivo [6,39], genetic variation in man, [40,41] while vascular intimal thickening and up – regulated iNOS has been described in mice [42]. These seemingly contradictory effects are probably explained in part by the different tissue situations and also by variation in the constituents of the cigarette smoke. This is an important factor in the preparation of the CSS and while CSS represents the aqueous phase of the stimulus, the gaseous portion may easily contain additional stimuli which we did not study. For these reasons investigators are often exposing cells in vitro to direct cigarette smoke rather than CSS alone to more accurately simulate the in vivo situation.

The major findings of this study were that CSS inhibited mast cell degranulation, production of NO by mast cells and tracheobronchial epithelial cells, as well as expression of the dominant NOS isoform by these cells. We had hypothesised that the NO_X_-rich CSS might exert a negative feedback mechanism upon NO release from these epithelial and mast cells. The addition of scavengers did not inhibit the effect, nor did SNP, a NO donor mimic CSS, thus disproving this hypothesis. In addition, NOS inhibitors did not affect the response, indicating that the endogenous cellular production of NO was not involved in the response to CSS. Nicotine appeared reduce the ability of the A549 cell line to generate NOx, but this was not dose-dependent and was not seen in other cell lines. The significance of this apparently idiosyncratic response is unclear.

The effects of CSS shown here could not be attributed to the pharmacological activity of nicotine, but may to be related to oxidative free radicals as they are inhibited by N-acetyl-L-cysteine [44]. NAC, an anti-oxidant, has been studied quite extensively for its ability to exert protective effects. Because of its SH group, NAC scavenges H_2_O_2 _(hydrogen peroxide), ^•^OH (hydroxyl radical), and HOCl (hypochlorous acid). In addition, NAC reduces cellular production of pro-inflammatory mediators [43]. CSS causes a reduction in NOS expression, and the mechanism would therefore seem to be at the level of the gene. The inhibition of the effects of CSS by NAC would appear to be congruent with the observations that NAC can reduce DNA adducts, clastogenic changes and other cellular toxic effects caused by mutagens and cigarette smoke in *in vitro *and in animal models, reviewed in De Vries 1993 [43]. These observations have led to the use of NAC in clinical trials in an attempt prevent or reduce the risk of recurrence of cancers by using NAC and other anti-oxidants [44]. This study did not use additional methods to confirm that the effect of CSS was via reactive oxygen species and NAC has complex attributes with actions other than by acting purely as an anti-oxidant, e.g. mucolytic activity, L-cysteine donation, and these could also play a role in modulating the effects of cigarette smoke [44].

## Abbreviations

airway epithelial cells, AEC; cigarette smoke solution, CSS; endothelial nitric oxide synthase, eNOS; inducible nitric oxide synthase, iNOS; N-acetyl-L-cysteine, NAC; normal human tracheal bronchial epithelial cells, NHTBE; nitric oxide, NO; polymerase chain reaction, PCR.
